# New Prognostic Score for the Prediction of 30-Day Outcome in Spontaneous Supratentorial Cerebral Haemorrhage

**DOI:** 10.1155/2015/961085

**Published:** 2015-01-08

**Authors:** Rita Szepesi, Ibolya Katalin Széll, Tibor Hortobágyi, László Kardos, Katalin Nagy, Levente István Lánczi, Ervin Berényi, Dániel Bereczki, László Csiba

**Affiliations:** ^1^Department of Neurology, University of Debrecen, Clinical Center, Debrecen 4032, Hungary; ^2^Division of Neuropathology, Institute of Pathology, University of Debrecen, Clinical Center, Debrecen 4032, Hungary; ^3^Hygiene and Infection Control Services, Kenézy Hospital, Debrecen 4031, Hungary; ^4^Department of Biomedical Laboratory and Imaging Science, University of Debrecen, Clinical Center, Debrecen 4032, Hungary; ^5^Department of Neurology, Semmelweis University, Budapest 1083, Hungary

## Abstract

*Aims*. The purpose of the present study was to evaluate predictors of outcome in primary supratentorial cerebral haemorrhage. Furthermore, we aimed to develop a prognostic model to predict 30-day fatality. *Methods*. We retrospectively analyzed a database of 156 patients with spontaneous supratentorial haemorrhage to explore the relationship between clinical and CT characteristics and fatal outcome within 30 days using multiple logistic regression analysis. The analyzed factors included volumetric data assessed by neuropathological and CT volumetry. A second CT scan in survivors or neuropathological ABC/2 volumetry in nonsurvivors was used along with the baseline CT to assess the growth index of haematoma. *Results*. Systolic blood pressure, serum potassium and glucose levels, platelet count, absolute and relative haematoma volumes, and presence and size of intraventricular haemorrhage statistically significantly predicted the fatal outcome within 30 days. Based on our results we formulated a six-factor scoring algorithm named SUSPEKT to predict outcome. *Conclusions*. After validation the SUSPEKT score may be applicable in general clinical practice for early patient selection to optimize individual management or for assessment of eligibility for treatment trials.

## 1. Introduction

Spontaneous intracerebral haemorrhage (ICH) accounts for 8% to 14% of all strokes and carries the highest mortality rate of major stroke subtypes [1]. The pharmacological and surgical interventions are less efficient than in case of ischaemic stroke [2]. Half of the mortality occurs within the first 2 days as a result of brain herniation [3]. Thirty-five to 52% of patients are dead by 1 month and only 20% of patients live independently at 6 months [4].

Predictors of mortality and functional outcome have been studied widely [5–7], but no treatment has shown a proven benefit so far. Accordingly, comprehensive analysis of the potential predictive factors of short term fatal outcome is of great importance. Reviewing the data of ICH patients admitted to the Neurology Department, University of Debrecen (UD), we retrospectively analyzed the relationship between clinical and CT characteristics and case fatality at 30 days.

The autopsy rate of our department is more than 90%; therefore we could incorporate also the results of brain autopsies into our analysis. This is a unique opportunity (compared with similar studies) in the era of declining brain autopsy rates [8]. Although all of our patients were immediately investigated by CT or MRI at acute admission (and repeated if required by the patient's deteriorating condition), we also had opportunity to analyze the results of their brain autopsies, agreeing with the Agency for Healthcare Research and Quality U.S. Department of Health and Human Services: “clinical diagnoses, whether obtained from death certificates or hospital discharge data, contain major inaccuracies compared with autopsy diagnoses” [9].

Numerous pathological events might occur in the brain of ICH patients (e.g., enlargement of haemorrhage, secondary brainstem bleeding) between the first and second imaging [10] or between the last CT and death. On the other hand, there are ethical and financial limitations of daily CT/MRI during the agony phase for estimating the “final” pathological findings of our patients. So, in our study we combined the classical* in vivo* (image-based) volumetric evaluations with the measurements performed on the same formalin-fixed brain slices after the death of patients. Taking advantage of this opportunity, the ABC/2 method was also applied, using the initial CT as a point of reference, to assess the growth index of the haematoma in the nonsurvivor group as a novel approach. To the best of our knowledge, no remarkable previous attempts have been made to use this method on cadaver brains with ICH.

Furthermore, we developed a new scoring system to predict the risk of death within 30 days after primary supratentorial ICH using variables found to be independently and significantly associated with outcome by multiple logistic regression analysis. All of these variables can practically be reported within the first few hours after admission. After validation, this scoring system has the potential to be useful for early classification of patients.

## 2. Subjects and Methods

### 2.1. Patient Population

Ethical permission for these investigations was granted by the Regional and Institutional Ethics Committee, University of Debrecen, Medical and Health Science Center. We retrospectively reviewed the data of 156 Caucasian patients (71 nonsurvivors and 85 survivors) with primary supratentorial ICH admitted to our Intensive Care Unit (ICU) in a 53-month period. The outcome was defined as 30-day fatality for any reason. All patients were older than 18 years of age and were transported to our ICU within 24 hours of stroke onset. If this point of time could not be ascertained, we used the last time when the patient was known to be well. All patients routinely underwent a baseline nonenhanced CT within 30 minutes of arrival, and the CT confirmed the ICH. A second CT was obtained on average on the 10th day after admission or when symptoms deteriorated. Exclusion criteria were traumatic ICH, subarachnoid haemorrhage (SAH), vascular malformation, tumour, haemorrhagic transformation of ischaemic stroke (on admission or any CT performed later), postthrombolytic haemorrhage in ischaemic stroke, infratentorial ICH, and primary intraventricular bleeding. We did not include subjects who had undergone neurosurgical evacuation or drainage. All patients were treated on specialized stroke units with multiparametric monitoring.

### 2.2. Data Collection

The following data were collected retrospectively from patients' clinical notes partly based on the Debrecen Stroke Database [11, 12]: sex, age, current smoking, excessive alcohol consumption, systolic and diastolic arterial blood pressure, and pulse rate at arrival. Laboratory parameters were collected from the initial blood sampling: serum sodium, potassium, glucose levels, sedimentation rate, haemoglobin, white blood cell (WBC) and platelet counts, liver and kidney function tests, and coagulation parameters.

### 2.3. CT Analysis

Image analysis was carried out retrospectively by two consultant neuroradiologists of our Department of Biomedical Laboratory and Imaging Science, who were blinded to the outcome. CT scans were performed on two 16-slice MDCT (multidetector computed tomography) scanners (GE CT/e Dual, GE Lightspeed 16; GE Medical Systems). Slice thickness was 5 to 10 mms for supratentorial and 2.5 to 4 mms for infratentorial regions. Images were transferred to an offline image processing workstation as DICOM (Digital Imaging and Communications in Medicine) files. Haemorrhage segmentation was carried out using the 3D Slicer software package developed by Brigham and Women's Hospital Surgical Planning Laboratory and MIT (Boston, Massachusetts, USA) [13]. This procedure allowed separation of the intracranial space from the skull and nonbrain structures; however, this method required verification and manual detachment of incorrectly labelled areas before performing volumetry with the built-in “Measurevol” module. The following variables were constructed: total intracranial volume; total haematoma volume; intraparenchymal haematoma volume; and intraventricular haematoma volume, each expressed as cm^3^. Additionally, relative volumes were defined as the ratio of total, intraparenchymal, and intraventricular haematoma volumes to intracranial volume yielding variables (without unit).

### 2.4. Neuropathological Analysis

Autopsies were performed within 48 hours after death. In our Neuropathology Laboratory, brains fixed in 10% formalin were cut into coronal slices. During the examination we verified the clinical diagnosis. Similarly to the* in vivo* diagnostic the ABC/2 method was used to estimate haematoma volumes [14, 15] based on the assumption that the volume of an intracranial haematoma can be approximated by an ellipsoid unless the haematoma is very irregular in shape. Ellipsoids can be described in terms of Cartesian coordinates using their three largest perpendicular axes [16]. Consecutive coronal slices were laid down with the posterior surface up, and maximal diameters of the haematoma on every slice were measured in mms: one in the horizontal direction and another perpendicular to that. These two largest diameters were used as A and B in the ABC/2 formula and were not necessarily on the same brain slice; each slice has been photo documented. Although slices were originally 10 mms thick, we measured the thickness of each slice at four different locations assuming that the brains may have shrunk during fixation. The precise thickness of a slice was obtained as the average of these measurements. The resulting numerical data were then summed to obtain the maximum diameter of the haematoma perpendicular to the previous ones, which was used as C in the ABC/2 formula. In cases when the haematoma did not fully penetrate a slice, that is, the first and last brain slice containing the bleeding, we measured the depth of the portion of haematoma by sticking a probe into it. Due to fixation in formalin and brain slicing in cases of ventricular extension it was not always exactly discernible which part of the haematoma was in the parenchyma and in the ventricles, respectively. Therefore the haematoma volume calculated by the ABC/2 method was equal to the sum of the intraparenchymal and intraventricular parts. Volumes of haemorrhages were converted from mm^3^ to cm^3^. In these cases the relative haematoma volume was calculated using the initial CT intracranial volumetry data (for methods see above).

### 2.5. Statistical Analysis

The growth index of haematomas was calculated in two steps according to the following logistic formulas (log indicates natural logarithm): *L* = log⁡(hpb2/(1 − hpb2)) − log⁡(hpb1/(1 − hpb1)), and growth  index = exp⁡(*L*)/(1 + exp⁡(*L*)) − 0.5, where hpb is haematoma per brain; hpb2 is ratio of total haematoma volume obtained by the second volumetry (follow-up CT scan in survivors, neuropathological ABC/2 method in nonsurvivors) to intracranial volume based on the follow-up CT (survivors) or the first CT (nonsurvivors); hpb1 is ratio of total haematoma volume to intracranial volume based on the first CT volumetry (survivors and nonsurvivors). Negative growth indices denote reduction of haematoma; positive growth indices denote haematoma expansion.

Variables were described using mean and SD (continuous variables) or category percentages (categorical variables) stratified for survivors and nonsurvivors. Associations between explanatory factors and the outcome of death within 30 days were evaluated using logistic regression. Curvatures in relationships were assessed and allowed for by transformation or by adding a squared term if this substantially improved model fit. Variables showing remarkable associations in unadjusted models were selected for multiple regression modeling unless they are collinear with each other, in which case elimination was carried out on grounds of clinical practicability. Left-out variables were assessed for their potential contribution by adding to the prefinal multiple model one by one and left in if found clinically remarkable or statistically significant. The final multiple logistic regression model was checked for goodness of fit using the Hosmer-Lemeshow test. The level of significance was set at *P* = 0.05. To create the scoring system, model-predicted probabilities of outcome were generated for all subjects, and statistics for the minimum, the 10th, 20th,…, 90th percentage points, and the maximum were derived from the resulting set of values. The coefficients of the final model were built into a spreadsheet-based calculation interface which places any patient, with given input data, in terms of probability-of-death percentage range, given the percentage points observed on our sample of patients. All analyses were performed using Stata Statistical Software (StataCorp. 2009).

## 3. Results

Of the 81 survivor patients, eight were excluded because they did not have a second CT. Of the 75 nonsurvivor patients we excluded two based on brain autopsy results: ruptured aneurysm of the middle cerebral artery and a metastatic tumour, respectively, had been confirmed. Twelve brains were unsuitable for volumetry due to fragmented, multilobar haematomas or having been damaged during removal from the skull, cases in which the ABC/2 method fails to accurately estimate haemorrhage volumes [17]. In addition, medical records were not sufficiently detailed in a total of nine cases. Thus, we analyzed the complete dataset of 59 nonsurvivor and 66 survivor patients.

The relationship between fatal outcome within 30 days and some selected discrete or continuous clinical variables is presented in Table 1. The relationship between age and greater odds of death was of borderline significance. Other variables, including sex, alcohol consumption, smoking, and pulse rate at admission, were not significantly associated with mortality. Higher systolic blood pressure at admission was a predictor of lethal outcome.

There was no statistical significance regarding sex, current smoking, excessive alcohol consumption, diastolic arterial blood pressure, and pulse rate at arrival. Laboratory parameters were collected from the initial blood sampling: serum sodium, potassium, glucose levels, sedimentation rate, haemoglobin, white blood cell (WBC) and platelet counts, liver and kidney function tests, and coagulation parameters. Of these, serum potassium concentration was significantly associated with fatal outcome: high and low levels outside the normal range both represented elevated odds of death. We identified higher serum glucose concentration and lower platelet count as further predictors of fatal outcome.

Tables 2 and 3 summarize the differences in CT characteristics and volumetric findings between survivors and nonsurvivors. The mean time from symptom onset to initial CT was strongly and inversely related to occurrence of death within 30 days. In the survivor group, the mean time between baseline and second CT was 10.7 (SD 5.05) days; in the nonsurvivor group, the mean time between symptom onset and death was 7.7 (SD 6.4) days. Differences between the two groups in absolute volumes of total and intraparenchymal haematoma on baseline CT were strongly significant, similarly to relative haematoma volumes. The growth index of haematoma was found to be a very strong predictor of the outcome. The early presence of intraventricular haemorrhage and its volume was also strongly associated with fatal outcome. There was an approximate 43% decrease in the mean volume of total haemorrhage from baseline to follow-up CT in survivors and a 54% increase from initial CT to death in the other group and, consistently, there was a 42% decrease in survivors and a 56% increase in nonsurvivors in relative total haematoma volume. Intraventricular extension of the haematoma was more frequent in the nonsurvivor group both on initial CT and on follow-up examinations.

### 3.1. The SUSPEKT Scoring System

The final multiple logistic regression model showed statistically significant associations between 30-day case fatality and a number of variables: serum glucose (*Su*gar); total haematoma volume (*S*ize); systolic blood* P*ressure; presence of intraventricular haemorrhage (*E*xtension to the ventricular system); and serum potassium level (*K*alium). Using these and adding age (life*T*ime) which was of borderline significance we developed the six-factor scoring system* SUSPEKT*. The SUSPEKT scoring system in use to make predictions after primary supratentorial ICH is demonstrated in Tables 4 and 5. Values of *x* represent means observed in the present study, hence the decimal fraction for intraventricular haemorrhage; *e* denotes Euler's number.

## 4. Discussion

Predictors of fatal outcome in primary ICH have been widely studied [6, 18–24]. However no reliable and widely used scoring system has been established as yet. Our aim was to assess a relatively simple, reproducible, cost-effective predictive scoring system by analysis of a wide range of clinical and epidemiological data. Our results show that systolic blood pressure, serum potassium and glucose levels, and platelet count independently and statistically significantly predict the 30-day fatal outcome in primary supratentorial ICH. Therefore, we propose a predictive scoring system for clinical outcome in ICH using six parameters (serum glucose; total haematoma volume; systolic blood pressure; presence of intraventricular haemorrhage; serum potassium level; and age) with the acronym SUSPEKT. In the subsequent paragraphs we discuss the individual components of SUSPEKT score and some of the other analyzed candidate parameters which did not show significant correlation with outcome prediction.

Of the laboratory parameters, we found serum glucose (*SU*) level on admission to be significantly associated with lethal outcome and this result is also supported by previous studies [25, 26], while others reported that, in ICH, hyperglycaemia is not a significant and independent outcome predictor [6, 18, 27]. Recently Lee et al. confirmed in stroke patients by magnetic resonance imaging and spectroscopy study that acute hyperglycaemia adversely affects stroke outcome [28].

We also found that absolute and relative haematoma volumes (“*S*” for size), haematoma growth index, and presence and size of intraventricular haemorrhage are predictors of death within 30 days after ICH. We recommend the use of absolute haematoma volume since it is easier to calculate and its predictive value is similar to relative volume. Haematoma volume is a well-known essential predictor of outcome in patients with ICH [5, 6, 18–20, 29–33].

According to previous findings, high systolic [34] and mean arterial [27] blood pressure (*P*) on admission are associated with outcome, and admission diastolic blood pressure is associated with larger ICH volume [35]. We found only systolic blood pressure to strongly predict 30-day fatal outcome.

Presence and size of intraventricular haemorrhage (“*E*” for expansion) are also strongly associated with outcome in both overall and supratentorial cerebral haemorrhages [18, 20, 21, 26, 36, 37]. Haematoma expansion is a strong determinant of both mortality and poor functional outcome after ICH [7]. Brott et al. found a trend toward higher mortality at 4 weeks in patients with haemorrhage growth [38].

We observed a previously unreported, very strongly significant association between serum potassium (“*K*”) level and 30-day lethal outcome, which was related to both high and low levels outside the normal range, although others found that higher dietary potassium intake is associated with lower rates of stroke [39]. Similar (clinicopathological) observation has been published earlier by our group on patients with haemorrhagic transformation [40].

Our result could be explained by the fact that high and low potassium levels are associated with morbidity and mortality also through cardiac effects: ventricular fibrillation and cardiac arrest. At extreme serum potassium levels death probably occurred because of cardiac dysfunction and not directly due to intracranial mass effect.

Age (“*T*” for lifetime) had a borderline predictive *P* value of 0.054 consistent with previous observations that the role of age in outcome after ICH is controversial. Some prior studies reported that age was significantly and independently correlated with fatal outcome, while others did not find this association [5–7, 18–20, 29–31, 41] or only for patients aged ≥80 years [6]. We found that the relationship between age and death within 30 days is of borderline significance.

We identified low platelet count as a predictor of fatal outcome and it is also reported that low platelet count and platelet dysfunction significantly correlate with haematoma growth [42]. Low haemoglobin showed no association with lethal outcome within 30 days. Although Kumar et al. [35] reported that anaemia and WBC count were associated with larger haematomas, none of these variables were predictors of 30-day mortality in concert with our results.

Time from symptom onset to initial CT was inversely and strongly associated with fatal outcome. This paradoxical relationship could be explained by cases of rapidly worsening complaints or severe signs caused by massive haemorrhages having presented to hospital sooner and, in contrast, slower response by patients experiencing mild impairment due to small haemorrhages.

There was no correlation between smoking and 30-day mortality. In a Japanese study smoking was an independent risk factor for death in both the ischaemic and haemorrhagic stroke [36], while other reports found no association [31, 37, 43]. Ikehara et al. reported that heavy alcohol consumption is significantly associated with increased mortality from haemorrhagic stroke for men [44]; however, in the present study we found no association between alcohol consumption and fatal outcome in ICH, which is in accordance with the results of Hansagi et al. [45].

Several prognostic models have been developed to predict fatal outcome after ICH, the most reliable and simplest being the ICH score by Hemphill et al. [6, 19, 22–24]. Prognostic scores limited to supratentorial haemorrhages are also known [18, 20, 21]. These studies focused on several factors: age [6, 19, 20, 22], sex [20], level of consciousness (GCS, NIHSS consciousness score) [6, 18, 19, 22], volume or location of haemorrhage [6, 18–20, 23], presence of intraventricular haemorrhage [6, 18–21, 24], presence of hydrocephalus on initial computed tomography scan [23], subarachnoid extension of haemorrhage [24], high NIHSS total score [22, 24], focal neurological deficit on admission [23], and pulse pressure [6, 24] were predictors of poor clinical prognosis; low temperature at admission and low NIHSS total score were predictors of good clinical outcome (modified Rankin score ≤ 2) [24]. Compared with the previously used prognostic score, undoubtable forcefulness of SUSPEKT score is that it includes serum potassium level which is a novelty and hyperglycaemia which can be corrected [2]. Another advantage of our scoring system is that it consists of only 6 parameters and except haematoma volume these can be managed and influenced by conservative (i.e., nonsurgical) therapeutic interventions.

Early prognostication after ICH is riddled with uncertainties. Given this and the self-fulfilling pessimistic general opinion of poor outcome, reliable early prognostication after ICH can help to avoid a fatalistic approach by the wider care team including family members and enables evidence based DNR (do not resuscitate) orders. Thus, deliberated guideline-concordant therapy is essential for all ICH patients [2]. Early prognostication helps clinicians to reach an objective opinion of predictable outcome.

Our findings are limited by some shortcomings. Prehospital deaths, exclusion of large, multilobar haematomas, and missing second CT in the survivor group may have led to possible selection bias.

## 5. Conclusion

We have demonstrated that systolic blood pressure, serum potassium and glucose levels, platelet count, absolute and relative haematoma volumes, haematoma growth index, and presence and size of intraventricular haemorrhage independently predict 30-day fatal outcome in primary supratentorial ICH. We developed the SUSPEKT score which after validation on large independent datasets may be applicable in clinical practice for patient selection to optimize individual management and for assessment of eligibility for clinical trials.

## Figures and Tables

**Table 1:** Summary of epidemiological and clinical data.

Characteristic	Survivors	Nonsurvivors	*P*
Age^*^	65.3 (11.83)	69.2 (13.48)	0.054
Male (%)	57.6	52.5	0.593
Alcohol (%)	34.8	23.7	0.462
Smoking (%)	25.8	18.7	0.303
Systolic blood pressure (mm Hg)	168.6 (32.93)	181.6 (34.86)	0.036
Diastolic blood pressure (mm Hg)	93.8 (17.85)	95.3 (18.53)	0.627
Haemoglobin (g/L)	140.0 (16.55)	137.5 (17.61)	0.408
WBCs (10^9^/L)^*^	9.5 (3.39)	9.3 (4.07)	0.292
Platelets (10^9^/L)	236.1 (87.34)	205.9 (77.31)	0.049
Glucose (mmol/L)	7.4 (2.86)	8.9 (4.64)	0.043
Potassium (mmol/L)^*^	4.1 (0.35)	4.0 (0.57)	<0.0001

The relationship between fatal outcome within 30 days and some selected discrete or continuous clinical variables. Age, sex, alcohol consumption, smoking, and pulse rate at admission were not significantly associated with mortality. High systolic blood pressure, abnormal serum potassium concentration, high serum glucose concentration, and lower platelet count at admission were predictors of lethal outcome. Serum potassium differences between survivors and deceased subjects did not manifest as different group mean levels because the latter group was a mixture of many hypo- and hyperkalaemic subjects whose values averaged out at a near normokalaemic level. WBC: white blood cell. Values are expressed as mean (SD) or percentages. ^*^ *P* value is from logistic regression including quadratic term.

**Table 2:** Baseline CT data.

Characteristic	Survivors	Nonsurvivors	*P *
Time from ictus to initial CT, hours	6.6 (7.3)	2.8 (2.6)	0.004
Total haematoma volume, cm^3^	16.3 (17.6)	57.8 (41.8)	<0.0001
Intraparenchymal haematoma volume, cm^3^	12.8 (14.2)	38.6 (30.6)	<0.0001
Intraventricular haematoma volume, cm^3^	3.6 (11.1)	19.2 (27.9)	0.002
Presence of intraventricular blood (%)	24.2	61.0	<0.0001
Relative total haematoma volume	0.012 (0.013)	0.043 (0.032)	<0.0001
Relative intraparenchymal haematoma volume	0.009 (0.010)	0.029 (0.023)	<0.0001
Haematoma growth index	−0.2 (0.16)	0.1 (0.18)	<0.0001

Differences in CT characteristics of survivors and nonsurvivors. The mean time from symptom onset to initial CT, growth index of haematoma volumes of total and intraparenchymal haematoma on baseline CT, and the early presence of intraventricular haemorrhage and its volume were strongly associated with fatal outcome. Values are expressed as mean (SD) or percentages.

**Table 3:** Total and relative haematoma volumes.

Characteristic	Survivors		Nonsurvivors	
	Baseline CT	Follow-up CT	Baseline CT	Pathology
Total haematoma volume, cm^3^	16.3 (17.6)	9.3 (11.2)	57.8 (41.8)	89.0 (56.45)
Relative total haematoma volume	0.012 (0.013)	0.007 (0.008)	0.043 (0.032)	0.067 (0.042)
Presence of intraventricular blood (%)	24.2	9.1	61.0	88.1

Differences in volumetric findings of survivors and nonsurvivors. Intraventricular extension of the haematoma was more frequent in the nonsurvivor group both on initial CT and on follow-up examinations. Relative volumes were defined as the ratio of total (intraparenchymal and intraventricular) haematoma volume to intracranial volume yielding unitless variables. Values are mean (SD).

**Table 4:** Example 1 for the use of SUSPEKT score.

Factor	Value in patient (*x*)	Coefficient (*b*)	Multiply *b* by *x* (*bx*)	
*SU*: serum glucose (mmol/L)	8.097	0.105504	0.854244	
*S*: relative total haematoma volume	0.027	68.94767	1.845598	
*P*: admission systolic BP (mm Hg)	174.728	0.003043	0.53161	
*E*: intraventricular blood (no = 0, yes = 1)	0.416	0.441198	0.183538	
*K*: serum potassium (mmol/L)	4.054	−19.2919	−78.2126	
Serum potassium squared ([mmol/L]^2^)	16.436	2.329607	38.28992	
*T*: age (years)	67.168	0.040057	2.690528	
Constant term	1	33.54228	33.54228	

Calculate by summing all *bx* values →			−0.2749	= *sbx *
Use the formula *e* ^*sbx*^/(1 + *e* ^*sbx*^) →			0.431706	= pr

The final multiple logistic regression model showed statistically significant associations with 30-day case fatality for a number of variables. An example of the SUSPEKT scoring system is presented. Values of x represent means observed in the present study, hence the decimal fraction for intraventricular haemorrhage; e denotes Euler's number. Enter patient's values and derive probability (pr) using the coefficients and follow the instructions.

**Table 5:** Example 1 for the use of SUSPEKT score.

0.036732	Minimum
0.095083	10th percentile
0.133933	20th percentile
0.190302	30th percentile
0.263309	40th percentile
0.376832	50th percentile
0.516875	60th percentile
0.769061	70th percentile
0.918411	80th percentile
0.975373	90th percentile
0.999965	Maximum

Table 5 illustrates a working example of the SUSPEKT scoring system. Refer pr (probability) (0.432, see Table 4) to the table: patient is between the 50th and 60th percentiles of the SUSPEKT learning dataset for probability of death.
